# From P3 to P2: Synthesis and Role of Morphology in Li-Substituted Layered Oxides for Na-Ion Batteries

**DOI:** 10.1021/acsenergylett.5c03108

**Published:** 2025-11-20

**Authors:** Mingfeng Xu, Prabhat Thapliyal, Jade Laurier, Rémi Tucoulou, Manfred Burghammer, Stefan Seidlmayer, Matteo Bianchini

**Affiliations:** † Faculty of Biology, Chemistry and Earth Sciences, University of Bayreuth, Universitätsstraße 30, D-95447 Bayreuth, Germany; ‡ Bavarian Center for Battery Technology (BayBatt), Weiherstraße 26, D-95448 Bayreuth, Germany; § CNRS, Univ. Bordeaux, Bordeaux INP, ICMCB UMR 5026, F-33600 Pessac, France; ∥ Laboratoire de Réactivité et de Chimie des Solides, CNRS-UMR#7314, Université de Picardie Jules Verne, F-80039 Cedex 1 Amiens, France; ⊥ European Synchrotron Radiation Facility (ESRF), F-38043 Cedex 9 Grenoble, France

## Abstract

Li-substituted P-type layered oxides with different morphologies and polymorphisms are investigated as positive electrode materials for sodium-ion batteries. *In situ* temperature-resolved X-ray diffraction (XRD) and spatially resolved synchrotron micro-XRD revealed the phase evolution during synthesis. For the first time, a P2 shell is shown to nucleate at the P3 particle surface, consistent with Na concentration gradients. The intermediate Li_2_MnO_3_, formed in the presence of Na_2_CO_3_, can be suppressed by using Na_2_O_2_ as Na source. Ni_1/4_Mn_3/4_(OH)_2_ is also tested as a precursor, allowing for faster P2 crystallization from P3. Three samples with different polymorphisms and morphologies are then synthesized and compared in terms of evolution of crystal and electronic structure during cycling. Galvanostatic intermittent titration technique (GITT) measurements are further employed to investigate the kinetics of the samples. A P2-polycrystalline material retains ≈90% capacity after 150 cycles and offers the best rate capability up to 10C, appearing as the most promising candidate for further development of high-performance layered oxides.

## Graphical abstract

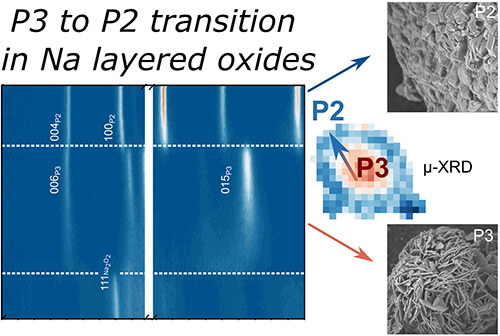

Sodium-ion batteries have emerged as a promising technology complementing lithium-ion batteries owing to lower cost and abundant raw materials.^1,2^ Recent commercialization progress further highlights their potential for practical applications, such as under low-temperature conditions.^3,4^ Among cathode materials, sodium layered oxides (Na_*x*_TMO_2_, TM = transition metal) stand out for their high theoretical specific capacity and scalable synthesis.^5^ These compounds comprise alternating Na and TM oxide layers, classified by the local coordination of sodium and the stacking sequence of oxygen.^6^ For example, in the common P2, P3, or O3 type, the letter refers to prismatic/octahedral Na sites and the number indicates a two-/three-layer stacking sequence of oxygen.^6,7^ P-type materials are particularly appealing because their prismatic pathways allow Na^+^ transport with a lower energy barrier as compared to their O-type counterparts.^8^

In our prior study, we investigated a series of Li-doped Ni/Mn-based P2 layered oxides (Na_5/6_Li_*y*_Ni_5/12–3*y*/2_Mn_7/12+*y*/2_O_2_, *y* = 2/18, 3/18, 4/18, 5/18).^9^ We found that modest Li-substitution enhanced the material’s structural stability and capacity retention. The optimal doping level was ≈1/9 Li per formula unit (/f.u.), which delivered a balanced capacity, cycling stability, and rate capability.^9^ The materials were synthesized by solid-state reaction from ball-milled carbonates and TM oxides, yielding large micron-sized particles referred to as “single-crystalline” morphology.^10,11^

In this work, to explore the possibility of further optimizing the material’s electrochemical performances, we use as an alternative precursor a coprecipitated hydroxide with well-defined particle morphology to yield distinctive “polycrystalline” products. Morphology is known to affect the electrochemical performance of layered oxide cathodes, although its influence can be complex. For Ni-rich NMCs, polycrystalline particles have been associated with either improved cycling stability due to reduced microstructural damage^12^ or accelerated fading from their higher surface area and particle cracking.^13^ Kim et al. also reported that the potential morphology-dependent parasitic surface reactions could cause capacity fade in P-type sodium layered oxides.^14^ Furthermore, precursor choice can alter both morphology and phase purity, enabling the formation of a pure P3 phase with composition similar to P2.^14^ Since P3 and P2 are polytypes with prismatic Na sites but distinct layer stacking and possibly Na^+^ transport behavior, we further studied the two structures during the synthesis process and compared their electrochemical performances.

Here, we systematically investigate three Li-doped layered oxides, their synthesis routes and the roles of morphology and polymorphism in their electrochemical performance when employed as positive electrode materials for sodium-ion batteries. Solid-state synthesis is a facile and widely adopted method for preparing P-type materials.^1,15–17^ We previously synthesized Li-doped P2 materials via solid-state reactions from finely ball-milled carbonates and oxides (Na_2_CO_3_, Li_2_CO_3_, NiO, and MnO_2_), yielding products with high purity (>95 wt %).^9^ However, employing the same precursors to obtain P3 materials of similar compositions has proven challenging, as P3 materials are only stable over a limited temperature range.^14,15,18^ Simply optimizing the calcination duration and temperature would inevitably result in the P2 phase. Even under a moderate calcination condition (625 °C, 4 h, Figure S1), P2 emerges and accounts for 16 wt % of the product.

To better understand the crystallization process and to tune it toward the effective synthesis of either pure P3 or P2 structures, we investigated it using both temperature-resolved and spatially resolved X-ray diffraction experiments. In Figure 1a,b, temperature-resolved *in situ* X-ray diffraction (XRD) patterns demonstrate the structural evolution pathways during the crystallization process using different precursors. First (Figure 1a), the established combination of the ball-milled Na_2_CO_3_, Li_2_CO_3_, NiO, and MnO_2_ powders was used. Two characteristic Bragg reflections of the precursors, 002 of Na_2_CO_3_ and 110 of MnO_2_, are marked by gray arrows. Upon temperature increase, Na_2_CO_3_ undergoes thermal expansion before decomposing.^19^ All precursors disappeared at ≈410 °C where the development of the 003 reflection of a P3 phase at 7.16° is observed. The crystallinity of the P3 phase increases progressively as the temperature increases. At ≈820 °C, P3 eventually transforms into P2 where the 015_P3_ completely disappears and a slight right-shift of the 00*l* is observed. The final P2 phase further crystallizes until the end of heating to 900 °C and during the temperature hold. While such ball-milled carbonate and oxide precursors allow a temperature window where only the P3 phase stabilizes, in practical synthesis with a scaled-up amount of materials and longer calcination time, it remains difficult to eliminate the P2 phase byproduct (Figure S1).

**Figure 1:**
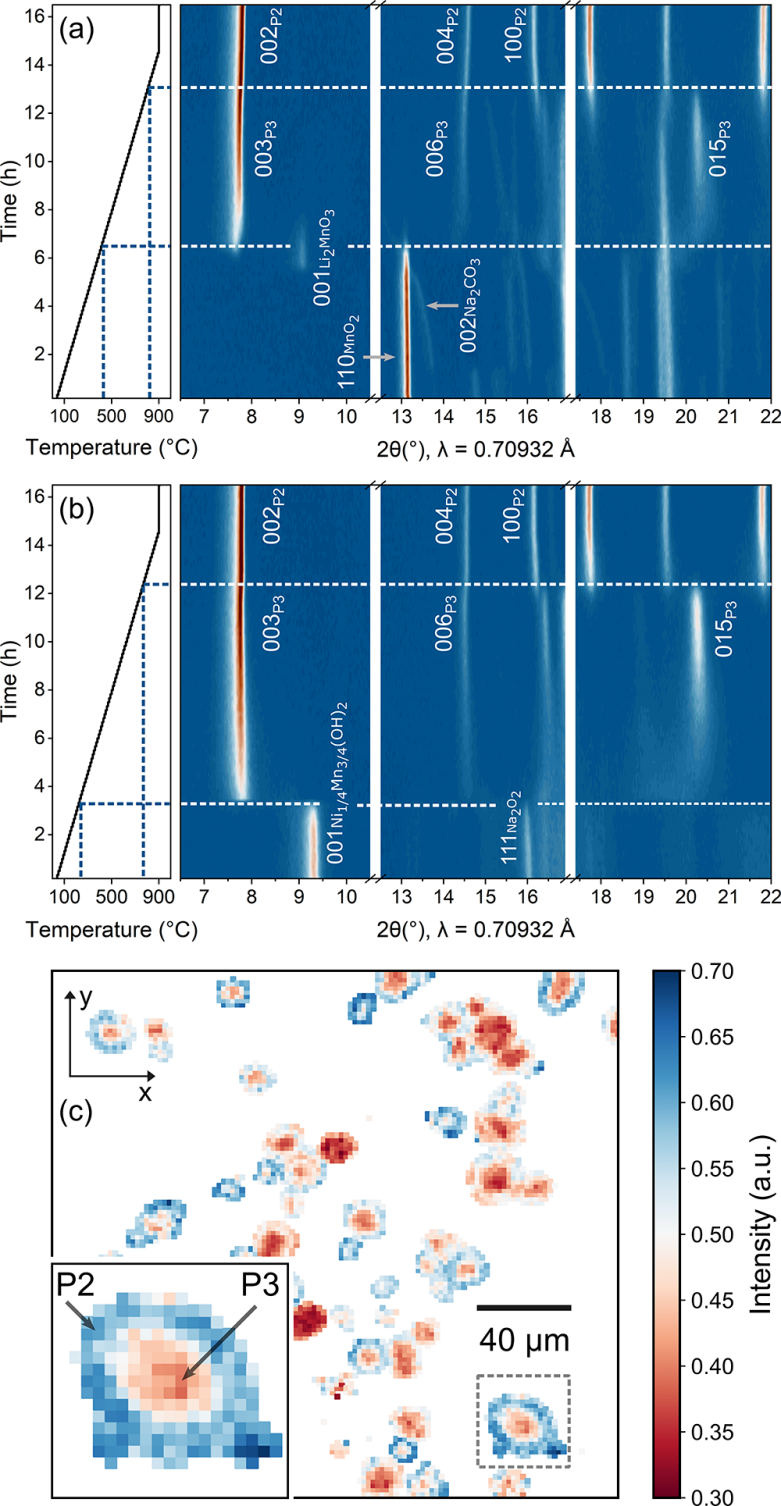
Temperature-resolved *in situ* XRD measurements during the synthesis using (a) ball-milled Na_2_CO_3_, Li_2_CO_3_, NiO, and MnO_2_ for the single-crystalline material; (b) ball-milled Na_2_O_2_, Li_2_CO_3_ and coprecipitated Ni_1/4_Mn_3/4_(OH)_2_ for the polycrystalline materials. (c) Micro-XRD image of the P2/P3 mixture with a spatial resolution of 2 μm × 2 μm per pixel. Reflections of 102_P2_ and 012_P3_ are selected to represent the spatial distribution of both phases. The axis of coordinates and intensity unit are arbitrary. Blue indicates higher intensity of 102_P2_, and brown indicates higher intensity of 012_P3_. The color bar denotes the fraction of the 102_P2_ intensity relative to the normalized total intensity of both phases. Note that the (near-)­white areas within the particles do not signify zero intensity but rather represent a similar and intermediate intensity of both phases. The visualization method is detailed in the Experimental Methods in the Supporting Information.

In an attempt to improve the purity of product P3, we tested an alternative combination of precursors, Na_2_O_2_, Li_2_CO_3_, and a coprecipitated Ni_1/4_Mn_3/4_(OH)_2_ (BASF SE) with a distinct secondary morphology. The SEM images of this precursor are presented in Figure S2. The primary particles are flake-shaped and grow together into spherical, well-dispersed secondary particles. The Na_2_O_2_ granular particles were ball-milled for 30 min to improve the reactivity, while Ni_1/4_Mn_3/4_(OH)_2_ was used as received. The mixed powders exhibit clear characteristic reflections of both materials (Figure 1b): 001 of Ni_1/4_Mn_3/4_(OH)_2_ and 111 of Na_2_O_2_. During the synthesis, these reflections disappear abruptly at ≈230 °C. An attempt to slow down the heating rate from 1 °C/min to 0.25 °C/min in the range of 230–240 °C witnessed no improvement in tracking this rapid transformation. The much lower decomposition temperature proves the superior reactivity of this precursor pair. Meanwhile, the P3–P2 transition takes place earlier at ≈770 °C. In general, both combinations of precursors yield a P2 structure without extra phases when reaching the high temperature region.

In our previous work, an intermediate phase Li_2_MnO_3_ was found during the synthesis of P2 materials with various lithiation levels.^9^ This Li_2_MnO_3_ phase is again observed at 2θ ≈9° in Figure 1a, coexisting early on with the P3 structure and residual precursors. One reason for the formation of Li_2_MnO_3_ ahead of Na layered oxides could be the lower melting point of Li_2_CO_3_ (720 °C^20^) vs Na_2_CO_3_ (858.1 °C^20^), suggesting it is thermodynamically favorable for Li_2_CO_3_ to react with other precursors at an earlier stage. In contrast, the combination of Na_2_O_2_, Li_2_CO_3_, and Ni_1/4_Mn_3/4_(OH)_2_ leads to no additional Li_2_MnO_3_ phase (Figure 1b), indicating that Na_2_O_2_ reacts faster than Li_2_CO_3_. This is consistent with the low melting point of Na_2_O_2_ (460 °C).^21^ Indeed, Na_2_O_2_ has a lower reaction energy relative to Na_2_CO_3_ in the synthesis of other Mn-based P2 layered materials.^15^ Additionally, a combination of Na_2_CO_3_, Li_2_CO_3_, and Ni_1/4_Mn_3/4_(OH)_2_ was also tested (Figure S3). In this case, P2 crystallizes at 800 °C, close to the P3–P2 transition temperatures of 820 and 770 °C found above. When using Ni_1/4_Mn_3/4_(OH)_2_, we consistently observe slightly lower crystallization temperatures for P2. In Figure S3, Li_2_MnO_3_ still forms before the complete decomposition of Na_2_CO_3_ and with reduced intensity, further proving that its formation is strongly influenced by the sodium source. On the other hand, the oxidation state of Mn in the precursors could also affect the development of Li_2_MnO_3_. Having Mn­(II) in Ni_1/4_Mn_3/4_(OH)_2_ but Mn­(IV) in MnO_2_, the latter could ease the formation of Li_2_MnO_3_ (also Mn­(IV)).

While *in situ* XRD measurements provide the overall phase information, the local distribution of the P2 and P3 phases during the crystallization remains an intriguing question. Various studies have reported on the intergrowth within single-crystalline layered materials, such as P2–P3,^22,23^ O3–P3,^24^ and P2–O3,^25^ but rarely on polycrystalline particles. To spatially visualize the biphasic P2/P3 growth within the polycrystalline particles, we resorted to synchrotron micro-XRD imaging. The biphasic sample was synthesized at 700 °C (Figure S4). In Figure 1c, the secondary particles exhibit a clear gradient core–shell feature with the P3 phase dominant in the core (brown) and the P2 phase in the shell (blue). A magnified inset evidently shows that the P3–P2 phase transition takes place from the outer toward the inner part in the sphere of a secondary particle, which to the best of our knowledge had not been observed before. Such a core–shell feature suggests that the P2 phase needs some external influence to nucleate from the P3 phase. Considering that in general throughout the literature P3 materials are reported to contain less sodium than P2 ones (as we also describe later), it is unlikely that P2 requires oxygen from the environment, as the transition metals are less oxidized. On the other hand, as shown in Figure 1a,b, P3 forms quickly but with a low sodium content; it requires time and higher temperatures for the remaining sodium salts at the interface to react with P3 and grow the P2 phase. This is also consistent with the fact that the thermodynamic driving force from P3 to P2 is expected to be extremely small.^15^

After clarification of the crystallization process, all samples were prepared through solid-state synthesis using optimized temperatures and precursor combinations, as detailed in the Experimental Methods in the Supporting Information. The usage of the coprecipitated Ni_1/4_Mn_3/4_(OH)_2_ not only facilitates control over the phase purity but also allows one to investigate the role of morphology. The samples are named P2-SC (P2 single-crystalline), P2-PC (P2 polycrystalline), and P3-PC (P3 polycrystalline). As expected, P2-PC and P3-PC preserved the morphological features of the precursor. Figure 2 displays the XRD patterns of all samples in their pristine state alongside particle images acquired by SEM. The stoichiometry of the metal elements was characterized by ICP–AES (Table S1). SEM–EDS (Figure S5a,c,e) confirms the homogeneity of the elemental distribution. We observe that the P2-SC particles are somewhat agglomerated, yet single grains can be well-distinguished (Figures S6 and S7); hence, we refer to them as “single-crystalline” for consistency with the literature.^26^ As shown in Figure S5, primary particles of P2-PC and P3-PC are smaller than those of P2-SC, as they retain morphological features inherited from the hydroxide precursor.

**Figure 2:**
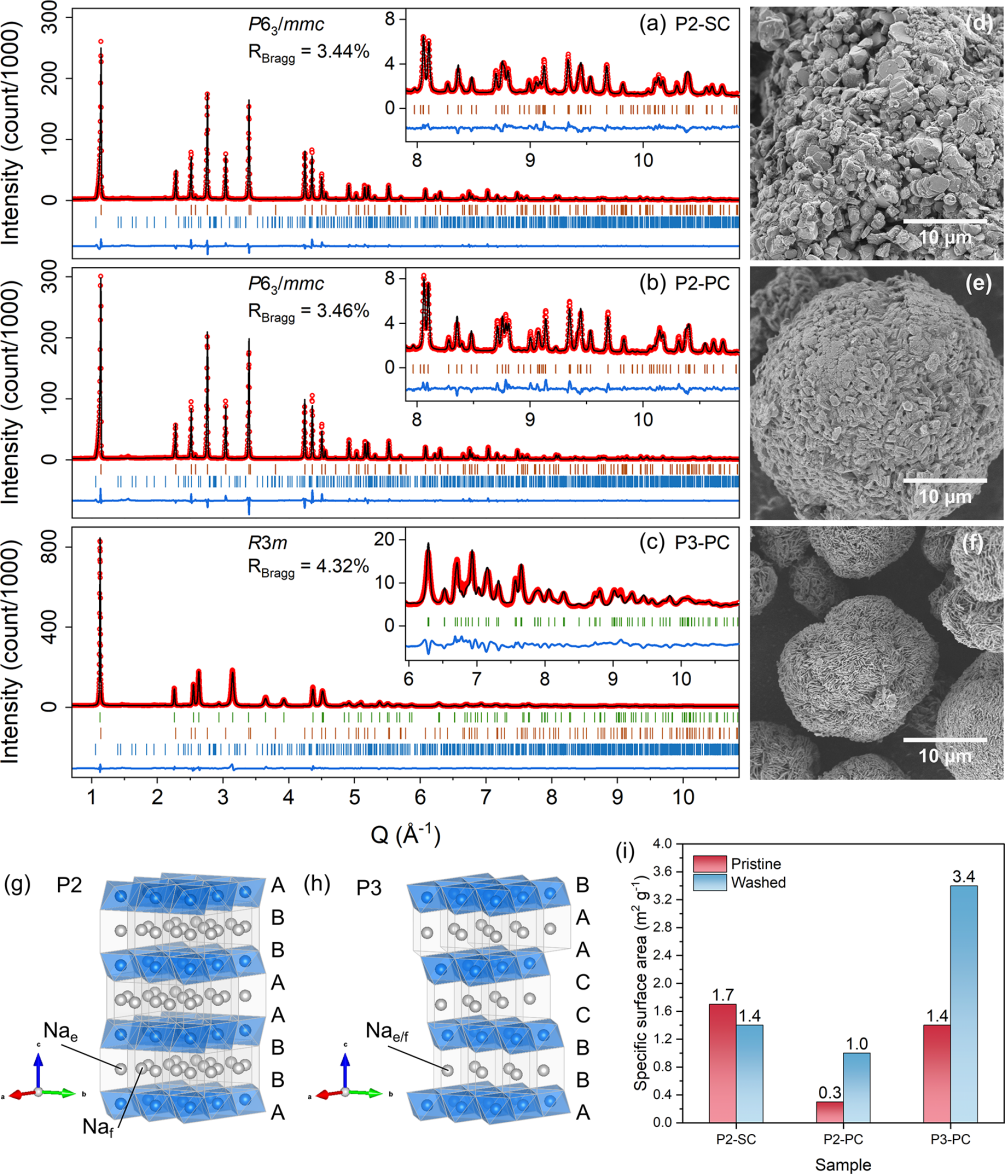
XRD patterns and Rietveld refinement results of (a) P2-SC, (b) P2-PC, and (c) P3-PC with a magnified high angular region. Brown, green, and blue bars are reference Bragg reflections of P2 (*P*6_3_/*mmc*), P3 (*R*3*m*), and Na_2_CO_3_ (*C*2/*m*), respectively. The P3-PC sample has a significantly larger scale of intensity due to the measurement performed at the synchrotron. Measurement details are provided in the Experimental Methods in the Supporting Information. Since the P2 samples were measured using a laboratory diffractometer with a different wavelength (0.70932 Å), the abscissa is converted to *Q*-space for all data sets. SEM images of (d) P2-SC, (e) P2-PC, and (f) P3-PC; acquisition details are provided in the Experimental Methods in the Supporting Information. (g) P2 and (h) P3 structures in an extended unit cell. In the P2 structure, there are two different Na sites: one in between the edges of the TMO_2_ octahedra on both sides, namely Na_e_; one in between the faces of the TMO_2_ octahedra on both sides, namely Na_f_.^6^ They are refined individually, as presented in Tables S2 and S3. In the P3 structure, Na ions (Na_e/f_) reside in between the edge of a TMO_2_ octahedron on one side and the face of another octahedron on the other side.^6^ The neighboring Na_e/f_ ions exhibit an inverse configuration of the edge and face of the TMO_2_ octahedra; thus, they are not differentiated in the refinement. (i) Specific surface area of all samples determined by BET analysis, before and after being washed by pure water.

The compositions and unit cell parameters were verified by Rietveld refinements (Figure 2 and Tables S2–S4). P2 samples demonstrate high Na content over 0.72/f.u. The Na amount in the P3 sample is relatively low (0.66/f.u.), likely due to a higher amount of unreacted residual Na_2_CO_3_ at lower synthesis temperature, which is indeed found as an impurity (Table S4). The different Na prismatic sites in P3 and P2 are shown in Figure 2g,h. Among all samples, P3 was synthesized at a relatively low temperature (625 °C) compared to the P2 ones (900 °C), resulting in lower crystallinity as indicated by the broader XRD peaks. A closer examination into the XRD pattern of the P3 is shown in Figure S8, which evidences the anisotropic broadening in certain reflections. Such broadening could be partly understood by the size contribution of the microstructure. The size model for Laue class 3̅*m* was applied and proven to noticeably improve the refinement (Figure S8a vs S8b, Figure S8c vs Figure 2c). The apparent sizes in all reflections are reported in Figure S9. In particular, 00*l* reflections suggest a larger apparent size (532 Å) than the average size (246 Å), indicating the elongation of the crystallite perpendicular to the *c*-axis, which is in good agreement with the flake-like primary morphology of the materials (Figures 2f, S6d, and S7d). Furthermore, a peak shift on broadened peaks like 015 and 107 may indicate the residual presence of stacking faults. On the other hand, both P2 samples, calcinated at higher temperature, exhibit bulkier primary particles, as shown in Figures 2, S6, and S7. In particular, the polycrystalline P2 shows the feature of partially fused primary particle grain boundaries, suggesting some intergrowth behavior, whereas the single-crystalline P2 particles tend to grow in a more isolated manner.

The different morphologies also lead to different specific surface areas, as determined by BET analysis (Figure 2i). Interestingly, the P2-PC sample displays significantly smaller specific surface area (0.3 m^2^ g^–1^) than the other two (1.7 m^2^ g^–1^ for P2-SC and 1.4 m^2^ g^–1^ for P3-PC). The low specific surface area of P2-PC is the result of sealed grain boundaries, i.e. most likely of residual alkali salts effectively filling surface porosity.^27^ P3-PC, also polycrystalline, has a higher surface area due to the smaller and clearly visible hexagonal primary particles stemming from the lower synthesis temperature; P2-SC instead shows the highest surface area due to the well separated grains. However, it should be kept in mind that the effective surface area can vary upon cycling.^13,27,28^ To infer the change of specific surface area that may occur, the samples were rinsed with water, and the BET was measured again (Figure 2i). This washing process removes the Na salts and opens up the surface porosity, analogous to what happens when the cathode material expands and contracts during cycling. Both polycrystalline samples experienced significant increases in specific surface area, almost tripling for P2-PC and doubling for P3-PC, suggesting a pronounced opening of their secondary particles. The specific surface area for P2-SC instead remains unchanged. In conclusion, after washing, P2-PC and P2-SC have comparable surface areas of 1.0 and 1.4 m^2^ g^–1^, while P3-PC reached 3.4 m^2^ g^–1^, which is significantly larger, likely to impact the subsequent electrochemical results.

P-type layered oxides tend to undergo severe phase transitions (P2–O2 ^9,29,30^ or P3–O3 ^31^) during electrochemical cycling, which can be effectively mitigated by doping inactive elements.^5,32,33^ We employed operando XRD during the first cycle for P2-PC and P3-PC electrodes to monitor their structural changes, as shown in Figure 3. For P2-SC, the operando synchrotron XRD was reported in our previous work.^9^ For both P2 and P3 samples, the most significant change occurs in the *c* parameter, which expands as Na ions deintercalate during charge, due to the decreased screening of interlayer repulsion between TM layers; conversely, during discharge, Na reinsertion restores the electrostatic screening so *c* decreases.^17,34^ The *a* parameter decreases in charge (and increases in discharge), primarily due to the oxidation/reduction of Ni^2+^, which lowers the overall TM ionic radii in the *ab*-plane.

**Figure 3:**
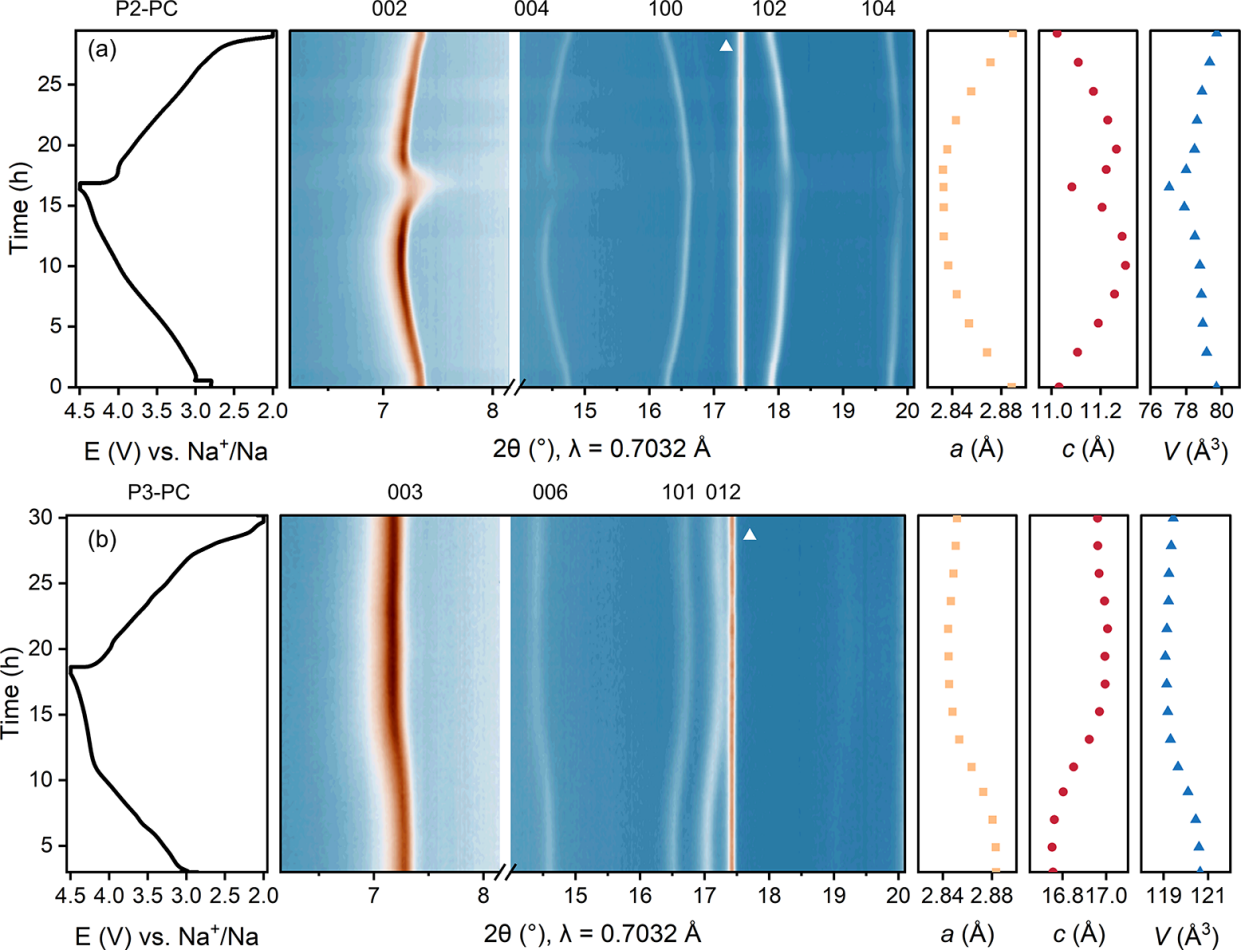
Structural change of (a) P2-PC and (b) P3-PC over the first electrochemical cycle. For each sample, from left to right are time-voltage curve, Bragg reflections, unit cell parameters *a* and *c*, and volume *V* obtained by LeBail fit. The peak from the sapphire capillary is marked by a triangle. Both cells were cycled at C/20 (1C = 200 mA g^–1^). The specific charge capacity at 4.5 V for P2-PC is 161 mAh g^–1^ and that for P3-PC is 155 mAh g^–1^, which suggests the volume changes of both materials at this point are comparable.

Given that both *c* and *a* vary, the unit cell volumes of both materials reduce during charge. In our previous work, P2-SC exhibited a volume change of 4.77% at 4.5 V (156 mAh g^–1^). In comparison, P2-PC delivers a comparable capacity, while the volume change is only limited to 3.34%. Notably, even after water rinsing, its specific surface area remains smaller than that of P2-SC (Figure 2i). Upon discharge to 2.0 V, the P2-PC’s unit cell returns to its original size. P3-PC exhibits even smaller volume contractions: 1.25% during charge and 0.99% relative to that in the OCV state. Li-doping suppresses phase transitions in P3; however, incomplete recovery of unit cell parameters in discharge suggests a nonideal structural reversibility of P3 compared to P2-PC.

The structural reversibility over long-term cycling is compared in Figure 4. By evaluating slow cycles at C/10 (5th and 158th), P2-PC demonstrates the best capacity retention of 90.5(7)%, followed by P2-SC with 85(1)% and P3-PC with 76.2(2)%. The initial Coulombic efficiency is 94.1(3)% for P2-PC, 84(1)% for P2-SC, and 80(2)% for P3-PC, again highlighting the superior reversibility of P2-PC in the first cycle. Figure 4b–d displays the galvanostatic cycling curves at C/10 for all samples over prolonged cycling. All samples deliver an initial charge capacity of over 135 mAh g^–1^ and discharge capacity of over 120 mAh g^–1^. Notably, P3 delivers the highest charge capacity of over 160 mAh g^–1^. P2-PC exhibits the smoothest cycling curves, suggesting superior structural stability. After five formation cycles, the capacity of P2-PC is rather stable, whereas those of P3-PC and P2-SC decay more quickly. A small slope above 4.2 V is observed in all samples (plateau), which is particularly evident during the first charge. For P3, this plateau extends longer than in P2 materials, potentially indicating an increased anionic redox contribution.^18,35^ To further understand this behavior, we measured XANES spectra for P3-PC during charge of the OCV to 4.5 V. The Ni and Mn K-edge XANES spectra in Figure S10 suggest that Ni was the only active TM during charge. As the material is close to full desodiation (*x* = 0.16, blue circle in Figure S10c), the Ni K-edge white line variation flattens (Figure S10d), suggesting Ni redox finishes contributing to the overall capacity.^9^ From this point, O-redox dominates and correspondingly contributes 20% of the total charge capacity. This is higher than that found in its P2 counterpart (10%),^9^ which explains its longer charging plateau at high voltages. Such a redox mechanism, where Ni-redox takes place at lower voltages, O-redox dominates at higher voltages, and Mn remains inactive, aligns with existing experimental^36^ and theoretical^37^ studies of P2 compounds with similar compositions and is consistent with our previous work on Li-doped P2 materials.^9^

**Figure 4:**
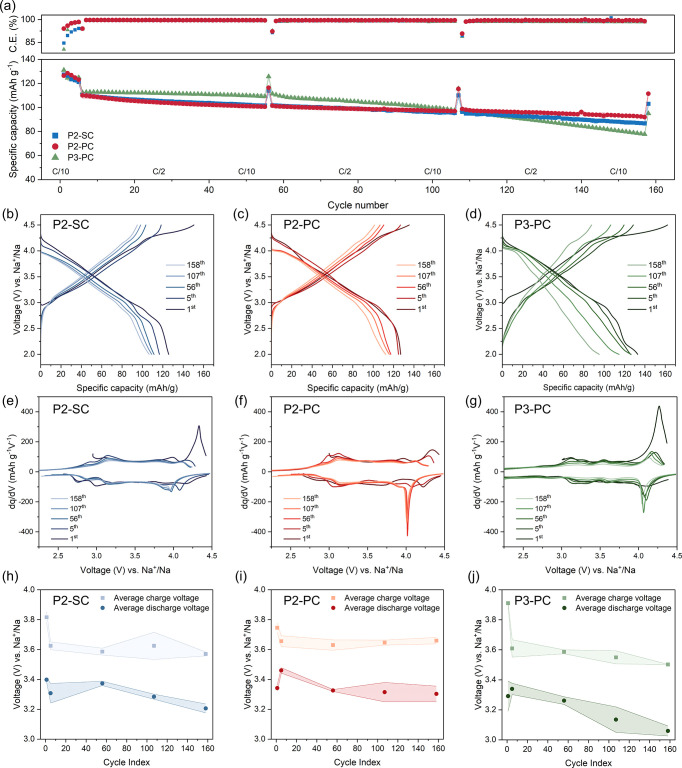
(a) Cycling stability of all materials in Na coin half cells. The Coulombic efficiencies of the 56th, 107th, and 158th cycles are not present as a cycle of C/10 was applied among cycles of C/2. (b–d) Galvanostatic charge/discharge curves, (e–g) d*q*/d*V* vs *V* curves during 1st, 5th, 56th, 107th, and 158th cycles at C/10 for all samples from one exemplary cell each. (h–j) Average voltages over the 1st, 5th, 56th, 107th, and 158th cycles at C/10 for all samples.

The d*q*/d*V* vs voltage curves are plotted in Figure 4e–g, corresponding to the voltage profiles in Figure 4b–d. Above 4.2 V during the first cycle, we again observe the O-redox in all materials, which is particularly intense in P3-PC. Upon extended cycling, the diminishing intensity of the O-redox feature points to its reduced contribution. The shifts of peak positions toward lower voltages over cycling also indicate a polarization increase likely due to degradation. Further insight into the polarization buildup is provided by the evolution of average charge/discharge voltages, as shown in Figure 4h–j. A clearly increasing polarization is observed for P3-PC, as is evident by the fast decrease in the average discharge voltage. Again, P3 experienced the greatest increase in the specific surface area, which may be conducive to faster degradation over cycling. In contrast, the P2 materials, particularly P2-PC, exhibit a more stable average charge and discharge voltage, indicating minimal polarization growth and eventually better specific capacity reversibility after long-term cycling at both C/10 and C/2 rates.

Over the medium-term cycling up to the 107th cycle, P3-PC maintains high capacity. Such behavior, where P3 outperforms P2 over early cycles, was also reported in other Mn-based P2 and P3 materials under similar rate conditions.^38^ However, the P3 structure undergoes the strongest irreversible anionic redox and has, by far, the largest surface area and content of Na salt residues, making it prone to side reactions. Such issues, though not significant in the initial cycles, are reflected in the overall lower Coulombic efficiency and accumulate over cycling, leading to increasing polarization, which suggests that P3-PC would perform even worse in full cells. Post-mortem XRD of P3-PC after 107 cycles discharged to 2.0 V in Figure S11 confirms its structural degradation with the presence of O3 phase.

Other than thermodynamic phase transitions and redox behavior, another key aspect to evaluate is the Na^+^ kinetics of all materials, especially given their different surface areas. To compare the kinetic behavior, the galvanostatic intermittent titration technique (GITT) was applied to examine Na^+^ diffusion during the first charge/discharge. The diffusion coefficients are presented against voltage in Figure 5 and against Na content *x* in Figure S12. Throughout the intermediate voltage range, all samples exhibit low overpotential and Na^+^ diffusion coefficients *D*_Na_ of ≈10^–9^ cm^2^ s^–1^, comparable to other reported P-type oxides,^31,39^ suggesting favorable Na^+^ kinetics. However, toward the end of charge, the overpotential increases. *D*_Na_ drops abruptly near 4.0 V (*x* = 0.3), coinciding with the onset of significant structural changes (interlayer collapse) observed by operando XRD (Figure 3). The drop is most pronounced in P3-PC, reaching even 5.5 × 10^–12^ cm^2^ s^–1^. This aligns with its strong redox peak in d*q*/d*V* at the same voltage (Figure 4g), which given the lack of clear phase transitions in operando XRD (Figure 3b) could be attributed to its stronger O-redox and potentially O_2_ release, leading to higher defectivity of the lattice, hindering Na^+^ transport. In P2 materials, while *D*_Na_ does not drop as severely at high voltages, the structural changes still impose a strong kinetic limitation on Na^+^ diffusion, likely due to the faulted structure often observed instead of a P2–O2 phase transition.^40,41^

**Figure 5:**
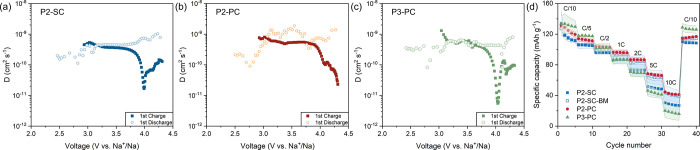
(a–c) Diffusion coefficients derived from GITT vs voltage of all samples. The cycling curves are present in Figure S13, and the respective diffusion coefficients were calculated based on the surface area of the electrode disc.^42^ (d) Rate capability of all materials, including an additional sample of ball-milled P2-SC-BM.

In terms of rate capability (Figure 5d), P2-PC delivers the highest capacities among all at 5C and 10C, likely due to faster Na^+^ transport and smaller primary particles. Since the rate capability of P2-SC materials could be influenced by its agglomeration state,^28^ we tested gentle mechanical grinding to deagglomerate the particles. The resulting particles (P2-SC-BM, Figure S14) display an improved rate capability, essentially identical with that of P2-PC, confirming the importance of a mild deagglomeration on the rate capability testing of single-crystalline P2.

In summary, we studied Li-substituted P-type layered oxides with different morphologies and polymorphisms as cathode materials for sodium-ion batteries. *In situ* temperature-resolved XRD and spatially resolved micro-XRD revealed the phase evolution during synthesis. P2 is shown to nucleate from the particle surface and grows inward, consistent with Na concentration gradients. The intermediate Li_2_MnO_3_, formed in the presence of Na_2_CO_3_, can be suppressed by using Na_2_O_2_ as Na source. The observed naturally occurring core–shell structure resembled highly engineered core–shell or concentration gradient NCM materials^43,44^ and may open new avenues for the design of advanced cathode materials. The synthesized materials P3-PC, P2-PC, and P2-SC were tested electrochemically. Operando XRD confirmed small volume changes during the first cycle in all materials, while XANES indicated that P3-PC undergoes more O-redox. GITT measurements showed similar diffusion coefficients (≈10^–9^ cm^2^ s^–1^) for all samples but with worse kinetics at high and low SOCs, in particular for P3-PC, which suffered from too high a surface area, leading to significant polarization growth upon cycling. In light of our experiments, purely P3 materials do not appear promising. On the other hand, P2 materials perform well. Long-term tests demonstrate that P2-PC has the best capacity retention (>90% capacity after 158 cycles), which is correlated to its lowest surface area even after washing. P2-SC follows very closely, but it shows slightly higher polarization buildup. Rate tests demonstrate that P2-PC also exhibits the best power capability up to 10C, yet this is very close to the values observed for P2-SC when properly deagglomerated. Our results indicate that, for appropriately engineered compositions (namely, with small volume variations and moderate O-redox), morphology itself plays a secondary role, while the surface area is the key factor determining performance.

## Supplementary Material



## Notes

Experimental methods and results, including materials synthesis methods, characterization methods, and electrochemical characterization methods; additional *ex situ* XRD patterns, SEM images (in different magnification levels), SEM–EDS results, temperature-resolved *in situ* XRD measurements, ICP–AES results, Rietveld Refinement details, XANES results of P3-PC, and additional GITT data (PDF)
