# Supplementary material for: From P3 to P2: Synthesis and Role of Morphology in Li-Substituted Layered Oxides for Na-Ion Batteries
Source: ACS Energy Lett. 2025 Nov 20;10(12):6347–55. doi: 10.1021/acsenergylett.5c03108 (PMC12706827; doi:10.1021/acsenergylett.5c03108)
Supplement: Supplementary file 1 [file nz5c03108_si_001.pdf]

Supporting Information

From P3 to P2: synthesis and role of morphology in Li-substituted layered oxides for Na-ion batteries

Mingfeng Xu[1, 2], Prabhat Thapliyal[1, 2], Jade Laurier[3,4], Rémi Tucoulou[5], Manfred Burghammer[5], Stefan Seidlmayer[1, 2], Matteo Bianchini[1, 2, *]

[1] Faculty of Biology, Chemistry and Earth Sciences, University of Bayreuth, Universitätsstraße 30, D-95447 Bayreuth, Germany

[2] Bavarian Center for Battery Technology (BayBatt), Weiherstraße 26, D-95448 Bayreuth, Germany

[3] CNRS, Univ. Bordeaux, Bordeaux INP, ICMCB UMR 5026, F-33600 Pessac, France

[4] Laboratoire de Réactivité et de Chimie des Solides, CNRS-UMR#7314, Université de Picardie Jules Verne, F-80039 Amiens Cedex 1, France

[5] European Synchrotron Radiation Facility (ESRF), F-38043 Grenoble Cedex 9, France

Experimental Methods

Materials Synthesis

Three layered oxides are explored in this study: a "single-crystalline" one with P2 structure (P2-SC), a "polycrystalline" one with P2 structure (P2-PC) and a "polycrystalline" one with P3 structure (P3-PC). All samples were prepared by solid-state synthesis and had the same target nominal composition. Stoichiometric precursors were ball-milled in zirconia jars by a SPEX-8000M ball-mill for 1 h. The sodium and lithium precursors are Na_2CO_3 (Alfa Aesar, 99.95%) and Li_2CO_3 (Alfa Aesar, 99.998%), respectively. Na_2O_2 (Sigma Aldrich, 97%, granular) is also used as alternative Na source. Given the ease of handling, however, Na_2CO_3 was used as a preferred source for the P2-PC synthesis. The nickel precursor NiO for P2-SC was obtained from decomposition of $Ni(OH)_2$ (Alfa Aesar, 99.999%) after heating it at 400 °C for 6 h in air. The manganese precursor was MnO_2 (Thermo Scientific, 99.9%). The nickel and manganese source for P2-PC and P3-PC is $Ni_{1/4}Mn_{3/4}(OH)_2$ obtained from a coprecipitation route (BASF SE). The P2-PC was calcinated at 900 °C for 12 h in ambient air with heating and cooling rate at 5 °C min^{-1}. The P2-SC was calcinated at 900 °C for 12 h in ambient air with heating and cooling rate at 5 °C min^{-1}, followed by a repeated calcination under the same condition for another 8 h to minimize the impurity. The P3-PC was synthesized at 625 °C for 12 h in

air with heating and cooling rate at 5 °C min^{-1}. To prepare deagglomerated P2-SC-BM, 300 mg of the P2-SC powders were ball-milled at 20 Hz for 20 min using a Fritsch Pulverisette 23 Mini Mill with two zirconia balls (3 g each). All synthesized products were transferred to an Ar-filled glovebox (200 B, MBraun, Germany; H_2O < 0.1 ppm, O_2 < 0.1 ppm).

Materials Characterization

Powder x-ray diffraction (XRD) patterns were measured on a STOE StadiP diffractometer (acquisition on sample powders packed in a borosilicate glass capillary of 0.5 mm diameter, in the 2θ range of 2.2–75.6° with a step size of 0.015°). The diffractometer was equipped with monochromatic Mo-Kα_1 (λ = 0.709319 Å) radiation and with a pair of MYTHEN 1K detectors. Crystallographic characterizations were achieved by Rietveld refinements with the FullProf Suite.[1,2] The stoichiometry of the metal elements was characterized by ICP–AES (inductively coupled plasma – atomic emission spectroscopy).

In situ temperature-resolved XRD measurements were performed in the same diffractometer by using a STOE INSITU HT2 high temperature reaction chamber. Precursors of each sample were filled in the center of a sapphire capillary of 1 mm diameter. The filled capillary was then placed in the reaction chamber and heated under ambient air. XRD patterns were collected in the 2θ range of 2.2–75.6° with a step size of 0.015° for 10 min at each temperature of interest.

High resolution scanning electron microscopy (SEM) images were acquired by a Zeiss Ultra plus microscope (Figures 2d, e, f, S6, S7, S15). An acceleration voltage of 3 kV was applied, and a secondary electron detector (SED) was used. SEM images for the coprecipitate precursor (Figure S2) and energy dispersive x-ray analysis (EDS) results (Figure S5) were acquired by a ThermoFischer Scientific Phenom ProX microscope. An acceleration voltage of 15 kV was applied, and an SED was used. ImageJ was used to determine the particle size distribution. At least 80 random primary particles for each material were selected and measured individually.

BET experiments were performed by a Quantachrome Instruments AUTOSORB-1 analyzer. For the pristine powders, approximately 200 mg of each sample was outgassed at 120 °C under vacuum for 24 h prior to the measurement at –196 °C using Kr adsorption. The same batch of powders were collected and washed in 30 mL pure water (for HPCL; VWR Chemical, Germany) and dried overnight under vacuum. The dried samples were again measured by the same protocol as above.

The micro-XRD imaging was performed at ID13, ESRF. The mixture powders were sealed between two Kapton tapes and spread sparsely to minimize overlap between individual particles. The samples were then scanned using an x-ray beam with a spatial resolution of 2 μm × 2 μm, which was the finest

achievable resolution given the instrumental limitations. While this size is still larger than some primary particles, it is sufficiently instructive to present the phase distribution within the secondary particles. Regions of 400 μm × 400 μm in the sample were selected for imaging, yielding 40,000 diffraction patterns per region, each corresponding to a single pixel in the x-ray image. To distinguish the two phases, we particularly focus on the reflections of 102_{P2} and 012_{P3}. All diffraction patterns were ultimately integrated into an overall XRD pattern, from which we select the Q-ranges of 102_{P2} and 012_{P3}, respectively. The spatial distribution of the two phases can then be mapped. Other reflection pairs at low Q-ranges (with good intensity), i.e., $002_{P2}/003_{P3}$, $004_{P2}/006_{P3}$, and $100_{P2}/101_{P3}$, are overlapping thus not differentiable, whereas 102_{P2} and 012_{P3} are distinct in orientations and maintain sufficient intensity, as suggested in Figure S4. For each phase, the intensity of the selected reflections at each pixel (each an XRD measurement) is normalized to the maximum across the entire scanned region (eqn. 1 and 2). A data filter of 15% of total intensity is used to screen out the background. In the end, the fraction of each phase at each pixel is calculated based on the fraction of 102_{P2} intensity relative to the total normalized intensity of both phases (eqn. 3).

$$I_{P2} = \frac{I_{102_{P2}}}{I_{102_{P2},max}} \geq 0.15 \text{ (eqn. 1)}$$

$$I_{P3} = \frac{I_{012_{P3}}}{I_{012_{P3},max}} \geq 0.15 \text{ (eqn. 2)}$$

$$I = \frac{I_{P2}}{I_{P2}+I_{P3}} \text{ (eqn. 3)}$$

Electrochemical Characterization

The positive electrodes were prepared by tape casting. 85 wt.% of active cathode materials, 7.5 wt.% of conductive carbon (Super P, Alfa Aesar, 99%), and 7.5 wt.% of binder solution were mixed in a centrifugal mixer (THINKY ARE-250). The binder solution is 7.5 wt.% PVDF (polyvinylidene fluoride) in NMP (*N*-Methyl-2-pyrrolidone, Sigma-Aldrich, 99.5%). The mixed slurries were casted on the aluminum current collector. The loadings of the electrodes are 6–10 mg cm^{-2}.

Electrochemical tests were conducted using 2032 coin cells. The half cells were assembled in an Ar-filled glove box (200 B, MBraun; H_2O < 0.1 ppm, O_2 < 0.1 ppm) with metallic sodium (from dry stick sodium; Thermo Scientific) as the counter and reference electrode, three layers of porous glass fiber separators (Whatman, GF/A) and 80 μL of electrolyte (1 M sodium hexafluorophosphate ($NaPF_6$) in equivalent amount of ethylene carbonate/dimethyl carbonate (EC:DMC = 1:1 vol.%) with 2 wt.% additive of fluoroethylene carbonate (FEC)). The galvanostatic cyclic measurements were performed on a temperature-controlled battery system (Arbin Instruments; 25 °C). The half cells were cycled

between 2.0 and 4.5 V *vs.* Na^+/Na^0 at specific current rates of 1C = 200 mA g^{-1}. GITT tests were conducted in the same half-cell configuration during the first cycle. The electrode loadings were 5.90 mg cm^{-2} for P2-SC, 6.10 mg cm^{-2} for P2-PC and 5.90 mg cm^{-2} for P3-PC, respectively. Charging was done at a C/20 rate for 15 min, followed by a relaxation time of 2 h. Discharging was done at a C/20 rate for 15 min, followed by a relaxation time of 4 h. The calculation of diffusion coefficients was done using a customized Python script based on the electrode surface area.[3]

(1) Rietveld, H. M. A Profile Refinement Method for Nuclear and Magnetic Structures. *J Appl Crystallogr* **1969**, *2* (2), 65–71.

(2) Rodríguez-Carvajal, J. Recent Advances in Magnetic Structure Determination by Neutron Powder Diffraction. *Physica B: Condensed Matter* **1993**, *192* (1–2), 55–69.

(3) Michael Häfner; Tobias König; Matteo Bianchini. Automating the Evaluation of Diffusion Coefficients from GITT Experiments. *Unpublished*.

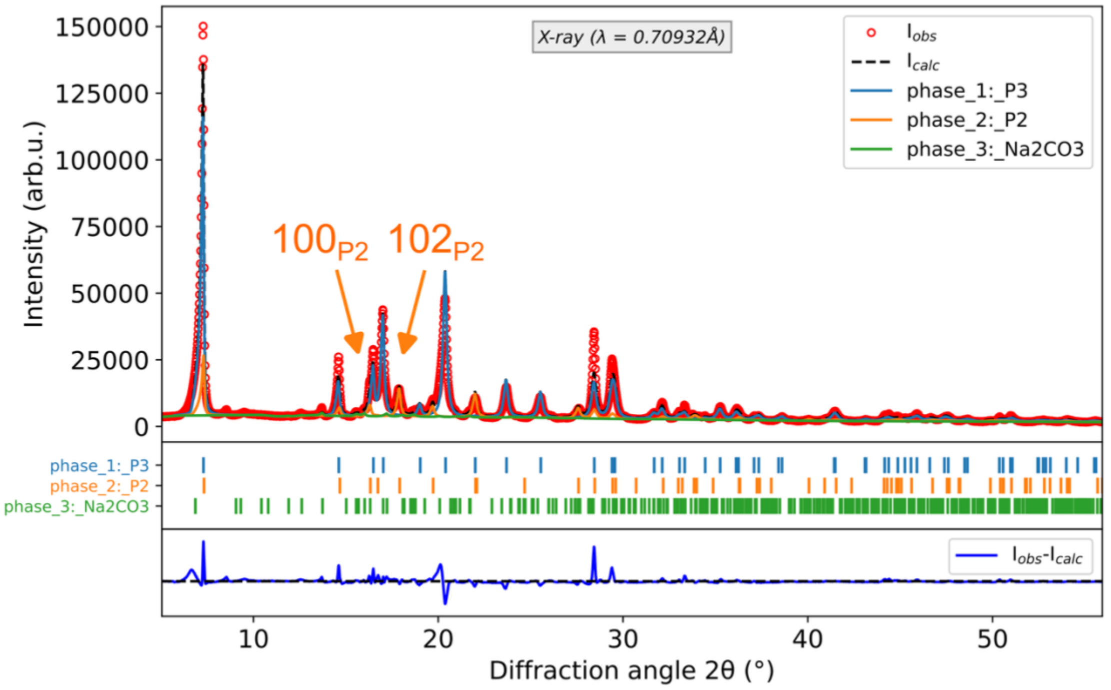

Figure S1. XRD pattern P3 structure synthesized from ball-milled Na_2CO_3, Li_2CO_3, NiO, MnO_2 at 625 °C for 4h. The short reaction time already resulted in the formation of significant amount of P2 (16.7(3) wt.%) and residual Na_2CO_3 (6.6(6) wt.%).

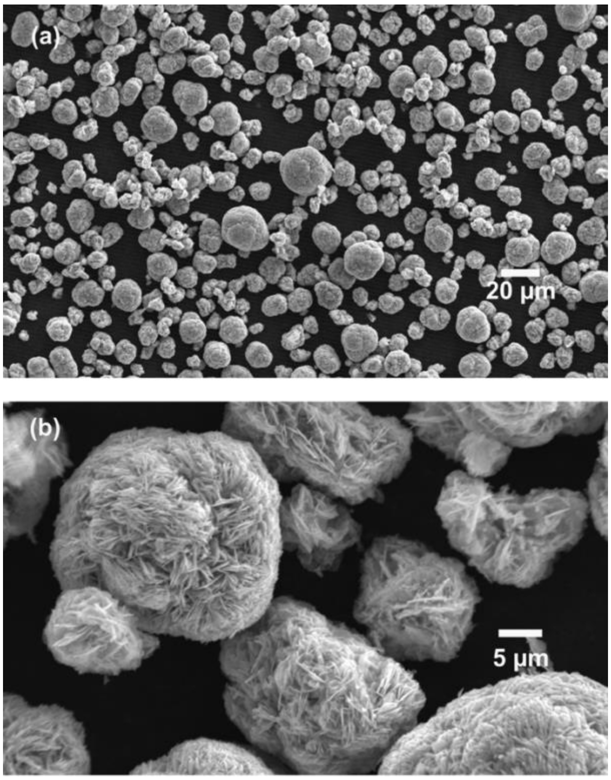

Figure S2. SEM images of the as-coprecipitated $Ni_{1/4}Mn_{3/4}(OH)_2$ precursor at (a) 2000× (b) 10000× magnification, acquired by a ThermoFischer Scientific Phenom ProX microscope.

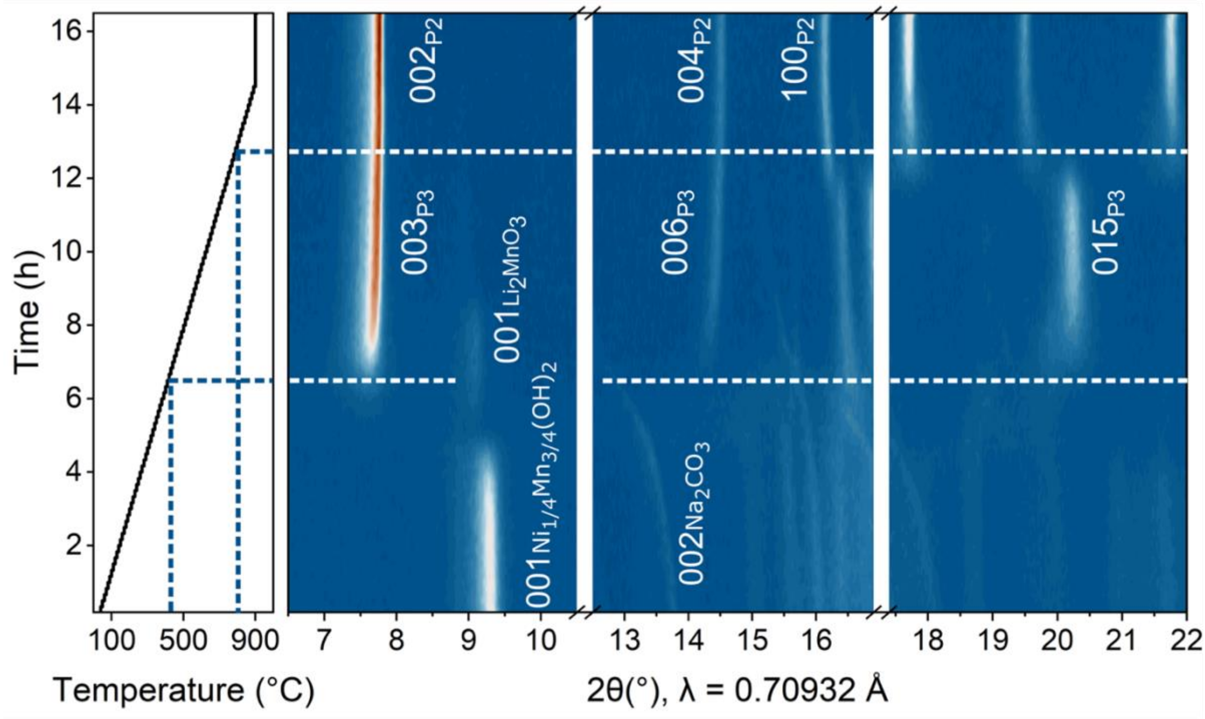

Figure S3. Temperature-resolved *in situ* XRD measurements during the synthesis using ball-milled Na_2CO_3, Li_2CO_3 and $Ni_{1/4}Mn_{3/4}(OH)_2$ for the polycrystalline materials.

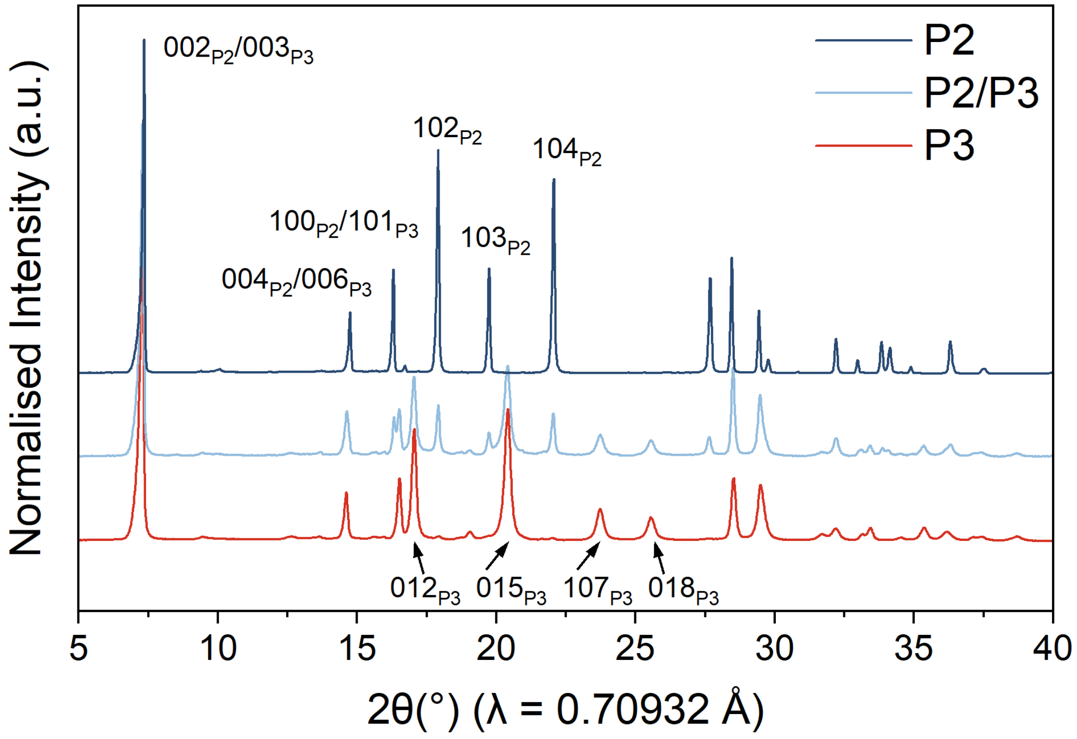

Figure S4. XRD patterns of polycrystalline P2 synthesized at 900 °C, P2/P3 mixture synthesized at 700 °C for 12 hours and P3 synthesized at 625 °C.

Table S1. Compositions of metal elements determined by ICP-AES, normalized to the formula Na_xMO_2.

Sample/Element	Na	Li	Ni	Mn
P2-SC	0.782(3)	0.115(0)	0.236(0)	0.649(1)
P2-PC	0.779(3)	0.112(2)	0.215(0)	0.673(1)
P3-PC	0.81(1)	0.104(2)	0.218(3)	0.678(4)

[1] The errors present are the standard deviation among three measurements.

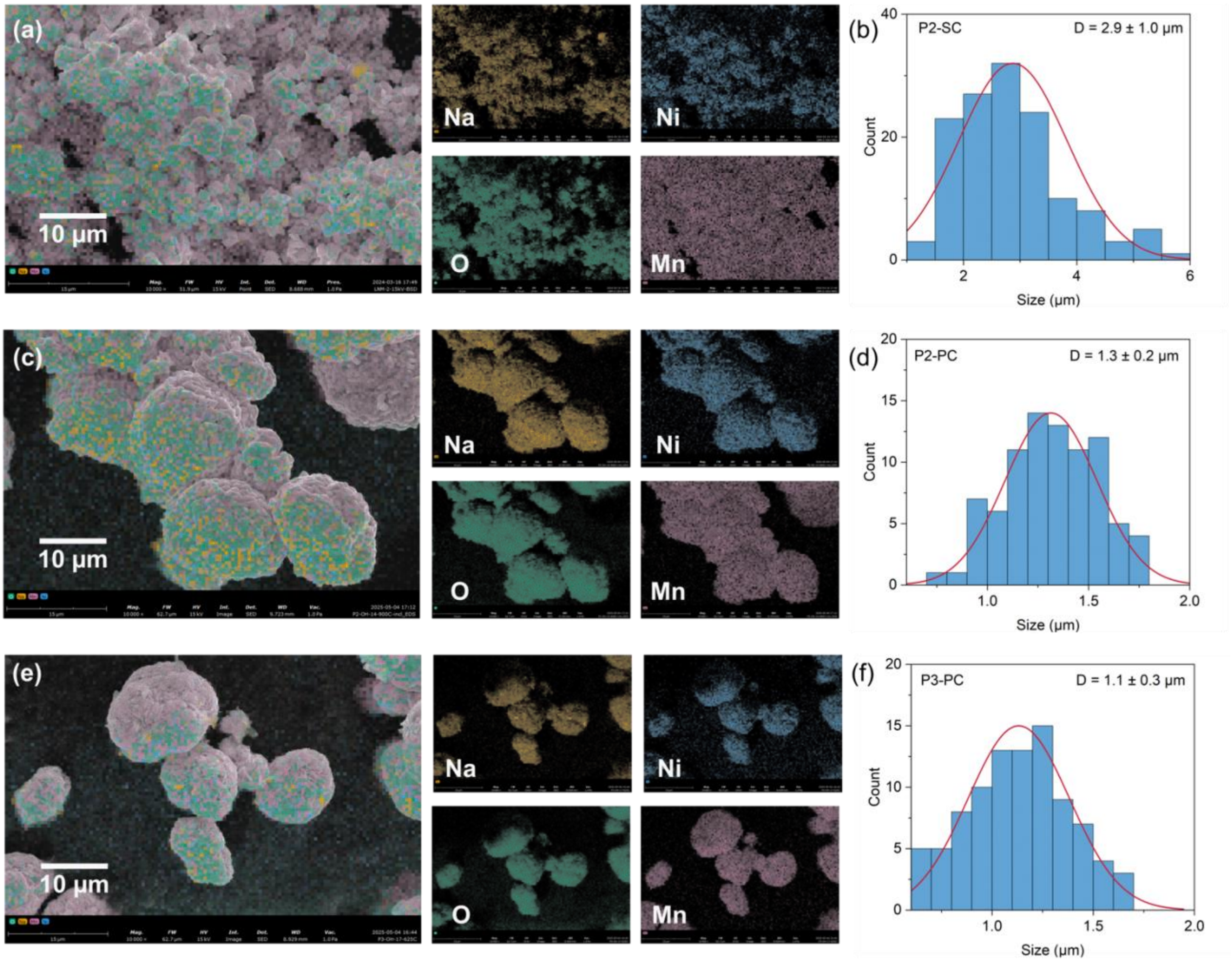

Figure S5. SEM–EDS mapping of (a) P2-SC, (c) P2-PC, (e) P3-PC, including elements of Na, Ni, O and Mn, with corresponding primary particle size distribution for (b) P2-SC, (d) P2-PC, (f) P3-PC, acquired by a ThermoFischer Scientific Phenom ProX microscope.

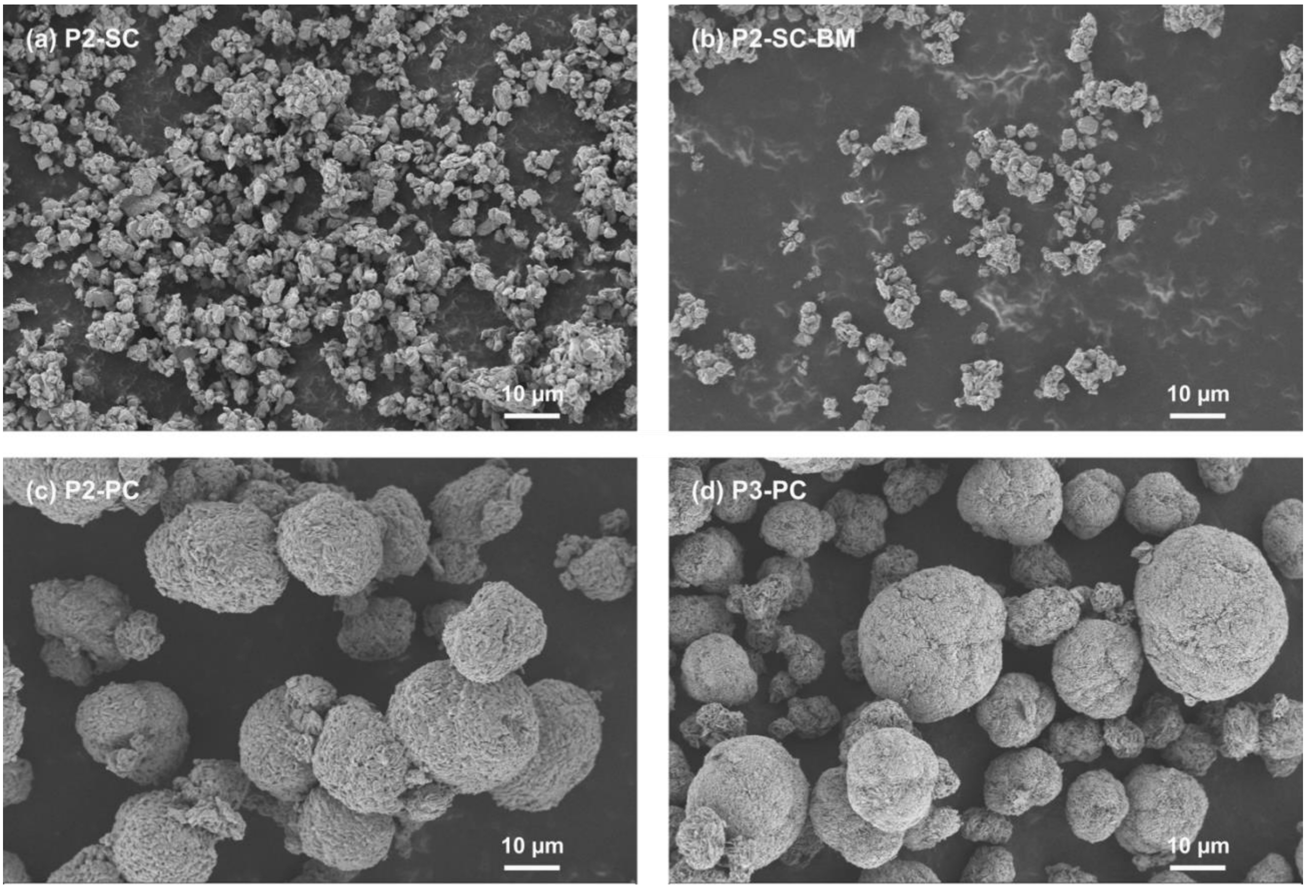

Figure S6. SEM images at 1000× magnification for (a) P2-SC, (b) P2-SC-BM (i.e. after ball milling for deagglomeration), (c) P2-PC, (d) P3-PC, acquired by a Zeiss Ultra plus microscope.

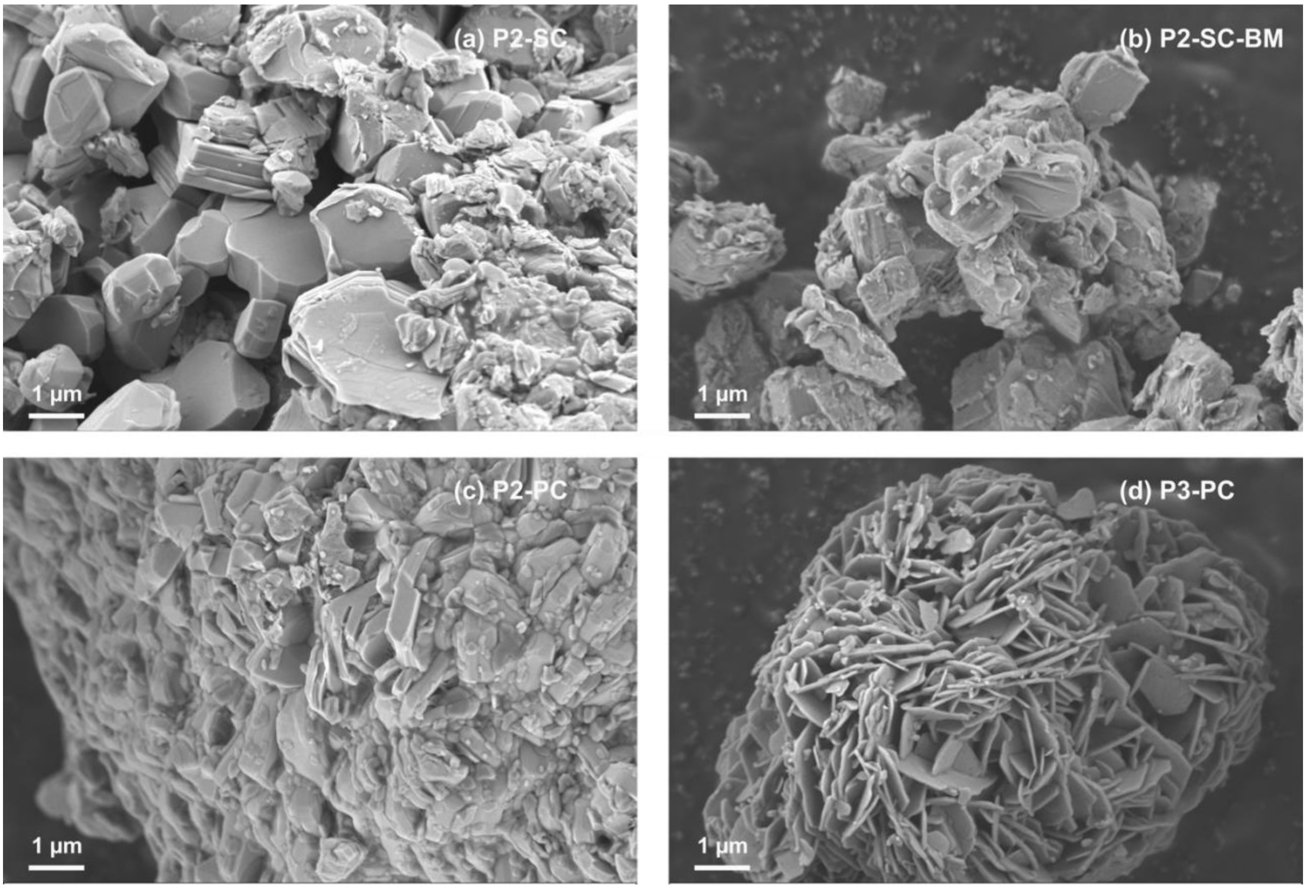

Figure S7. SEM images at 10000× magnification for (a) P2-SC, (b) P2-SC-BM (i.e. after ball milling for deagglomeration), (c) P2-PC, (d) P3-PC, acquired by a Zeiss Ultra plus microscope.

Table S2. XRD Rietveld Refinement of powder P2-SC using $P6_3/mmc$

$Na_{0.751(6)}Li_{0.115}Ni_{0.236}Mn_{0.649}O_2$				$P6_3/mmc$, $Z = 2$		
$a = b = 2.89004(1)$ Å				$R_{Bragg} = 3.44\%$		
$c = 11.02815(9)$ Å				$R_{wp} = 8.18\%$		
$V = 79.770(1)$ Å^3				$\chi^2 = 32.0$		
**Atom**	**Wyck. sites**	***x/a***	***y/b***	***z/c***	B_{iso}	**Occ.**
O	4*f*	1/3	2/3	0.0938(1)	0.76(3)	1.000
Li	2*a*	0	0	0	0.248(8)	0.115
Ni	2*a*	0	0	0	0.248(8)	0.236
Mn	2*a*	0	0	0	0.248(8)	0.649
Na_f	2*b*	0	0	1/4	4.1(2)	0.262(4)
Na_e	2*d*	1/3	1/3	3/4	2.3(1)	0.489(4)

[1] The occupancy in TM sites is fixed according the ICP-AES results, same as in Tables S3 and S4.

[2] Phase 1: P2, 96.9(4) wt.%. Phase 2: Na_2CO_3, 3.0(2) wt.%.

Table S3. XRD Rietveld Refinement of powder P2-PC using $P6_3/mmc$

$Na_{0.722(6)}Li_{0.112}Ni_{0.215}Mn_{0.673}O_2$				$P6_3/mmc$, $Z = 2$		
$a = b = 2.88516(1)$ Å				$R_{Bragg} = 3.46\%$		
$c = 11.04210(6)$ Å				$R_{wp} = 9.18\%$		
$V = 79.602(1)$ Å^3				$\chi^2 = 41.2$		
**Atom**	**Wyck. sites**	***x/a***	***y/b***	***z/c***	B_{iso}	**Occ.**
O	4*f*	1/3	2/3	0.0933(2)	0.64(3)	1.000
Li	2*a*	0	0	0	0.178(8)	0.112
Ni	2*a*	0	0	0	0.178(8)	0.215
Mn	2*a*	0	0	0	0.178(8)	0.673
Na_f	2*b*	0	0	1/4	3.2(2)	0.242(4)
Na_e	2*d*	1/3	1/3	3/4	2.1(1)	0.480(4)

[1] In P2-PC, Phase 1: P2, 98.2(5) wt.%. Phase 2: Na_2CO_3, 1.8(2) wt.%.

Table S4. XRD Rietveld Refinement of powder P3-PC using $R3m$

$Na_{0.664(3)}Li_{0.104}Ni_{0.218}Mn_{0.678}O_2$				$R3m$, $Z = 3$		
$a = b = 2.\ 87846\ (2)$ Å				$R_{Bragg} = 4.32\%$		
$c = 16.7048(3)$ Å				$R_{wp} = 7.32\%$		
$V = 119.865(2)$ Å^3				$\chi^2 = 635$		
**Atom**	**Wyck. sites**	***x/a***	***y/b***	***z/c***	B_{iso}	**Occ.**
O1	3*a*	0	0	0.3986(4)	0.74(3)	1.000
O2	3*a*	0	0	0.6067(4)	0.74(3)	1.000
Li	3*a*	0	0	0	0.63(9)	0.104
Ni	3*a*	0	0	0	0.63(9)	0.218
Mn	3*a*	0	0	0	0.63(9)	0.678
$Na_{e/f}$	3*a*	0	0	0.8303(2)	4.1(1)	0.664(3)

[1] Phase 1: P3, 95.1(3) wt.%; Phase 2: Na_2CO_3, 4.2(2) wt.%; Phase 3: P2, 0.79(6) wt.%.

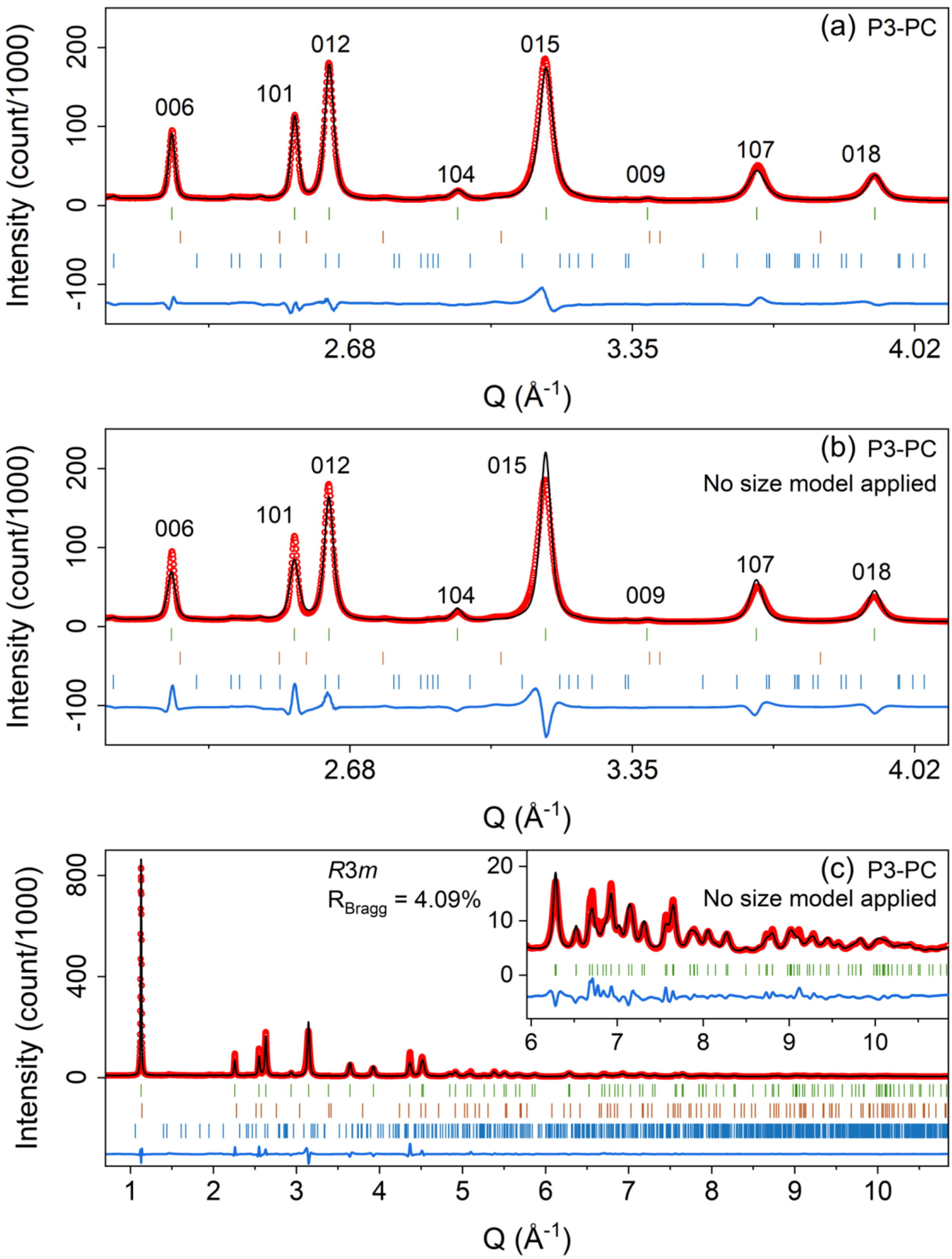

Figure S8. Rietveld refinements of the P3-PC (a) in the *Q*-range of 2.1–4.1 Å^{-1} using an anisotropic size broadening model. (b) in the *Q*-range of 2.1–4.1 Å^{-1} without using an anisotropic size broadening model. (c) in the whole *Q*-range measured.

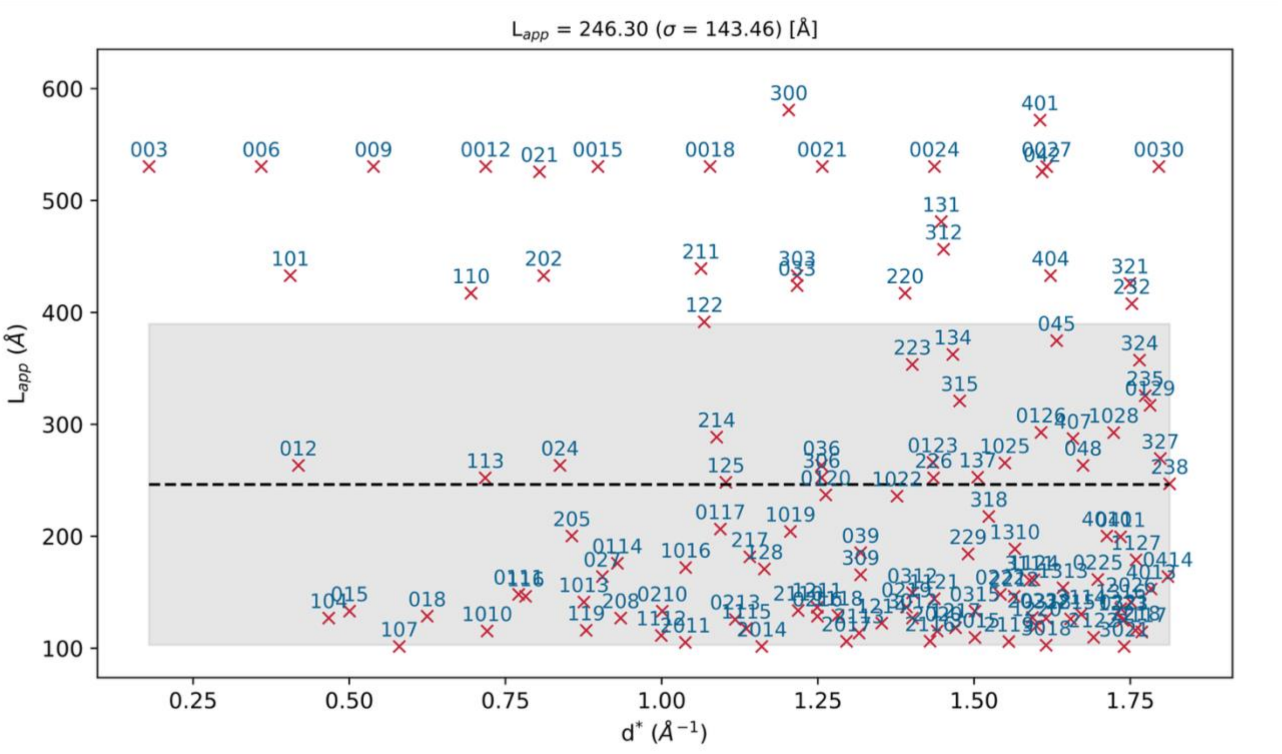

Figure S9. Apparent crystallite size obtained from individual Bragg reflections in phase P3. The grey area represents the standard deviation of the average apparent size: 246.30 ± 143.46 Å.

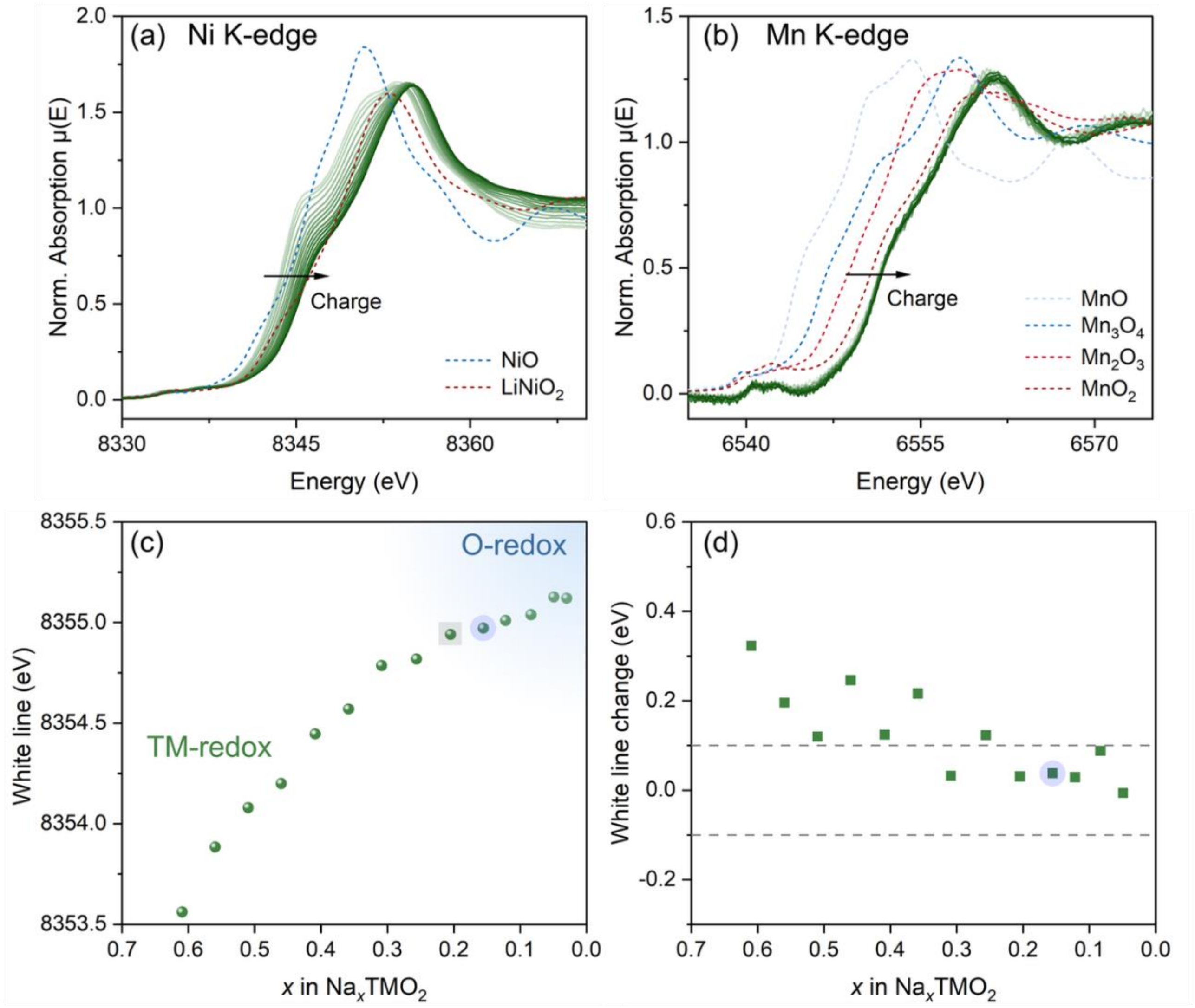

Figure S10. Operando XANES spectra of P3-PC during charge from 2.0 V to 4.5 V, (a) Ni K-edge, (b) Mn K-edge. (c) Ni K-edge evolution according to white lines shift during charge. The circled point is the beginning of O-redox based on the white line energy change; the grey square specifies the beginning of O-redox based on the Ni content. (d) White line change. Gray dashed lines are thresholds of 0 ± 0.1 eV. When at least two consecutive points lie within this band, the white line change is considered to flatten with minor oscillation, indicating Ni-redox almost finishes contributing.

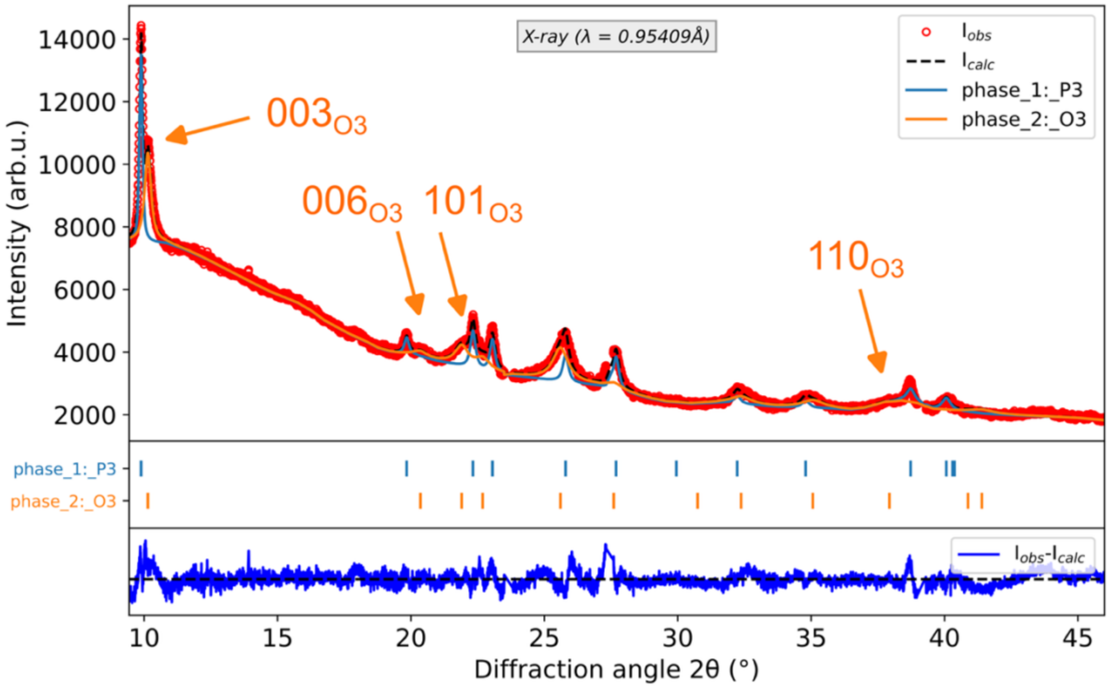

Figure S11. Post-mortem XRD pattern of the P3-PC electrode after 107 cycles discharged to 2.0 V. LeBail fit was performed to confirm the presence of the minor O3 phase.

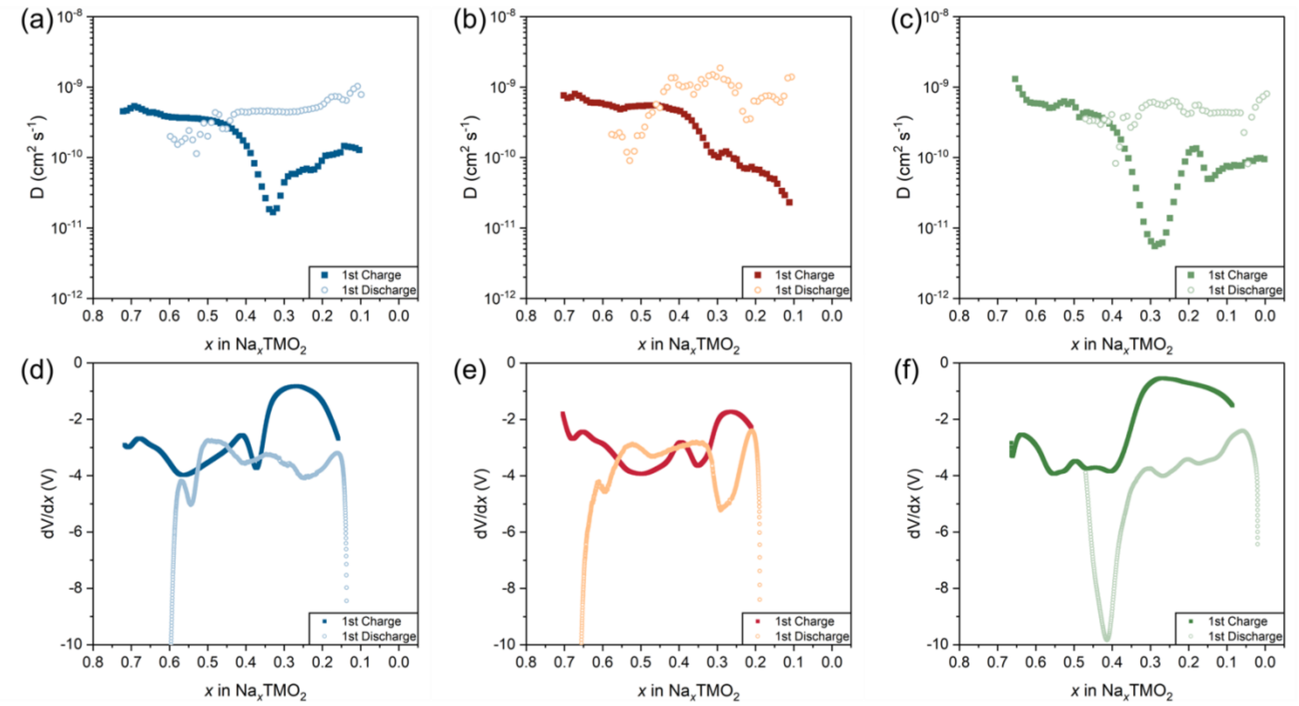

Figure S12. Diffusion coefficients derived from GITT *vs.* Na content x of (a) P2-SC, (b) P2-PC, (c) P3-PC, and accordingly the dq/dV *vs.* x curves of (d) P2-SC, (e) P2-PC, (f) P3-PC. Note that the GITT measurements were tested at C/20 while the other regular measurements were tested at C/10, hence the capacity or the Na content x between the panels up and down for each sample are not perfectly comparable. The absolute dV/dx values are less physically significant as they strongly depend on the numerical differentiation interval (in this case, for all samples, every 100 data points were used to compute the differences).

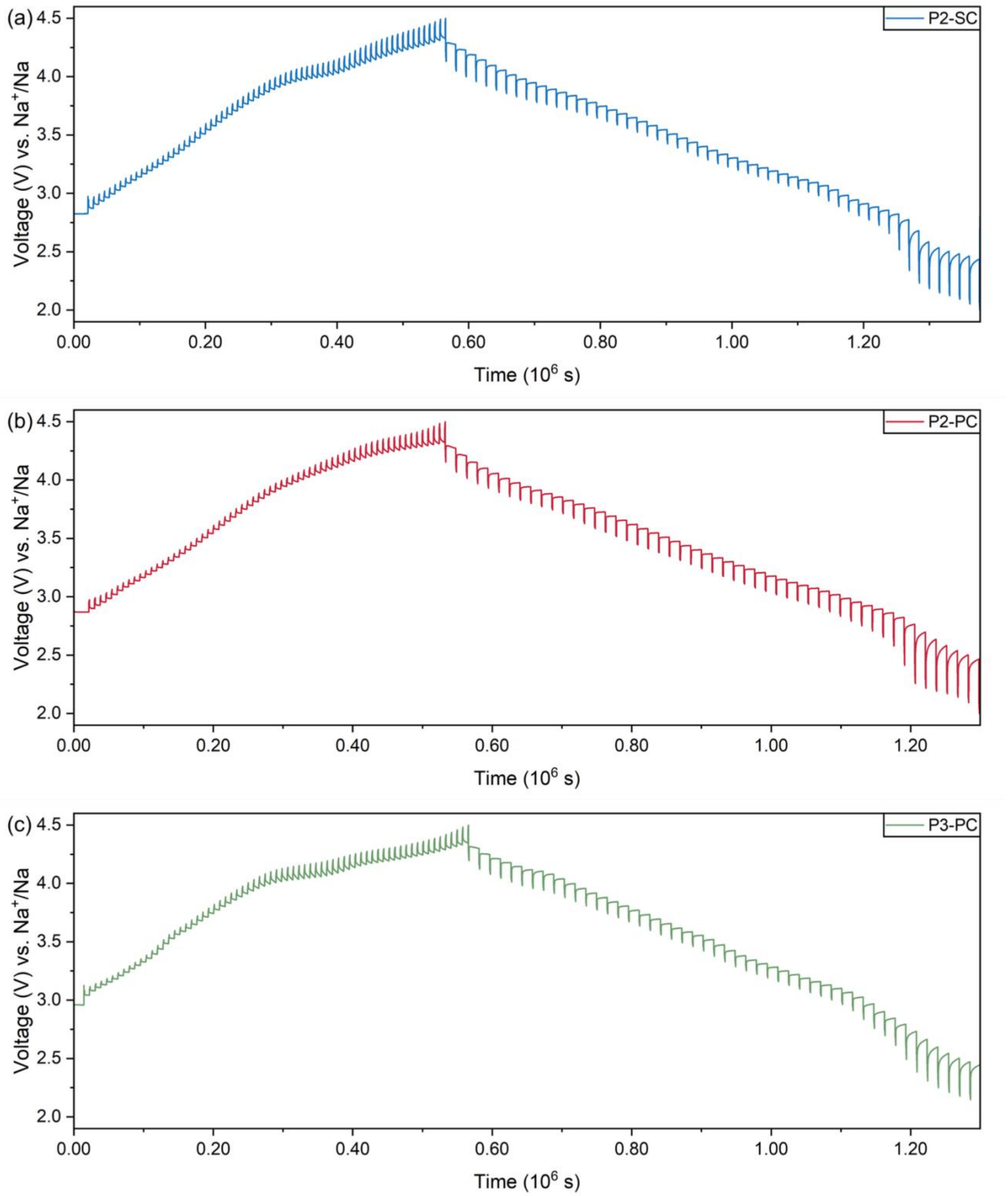

Figure S13. Galvanostatic intermittent titration technique applied to the half-cells for all samples during their first and second cycle. Charging was done at a C/20 rate for 15 min, followed by a relaxation time of 2 h. Discharging was done at a C/20 rate for 15 min, followed by a relaxation time of 4 h.

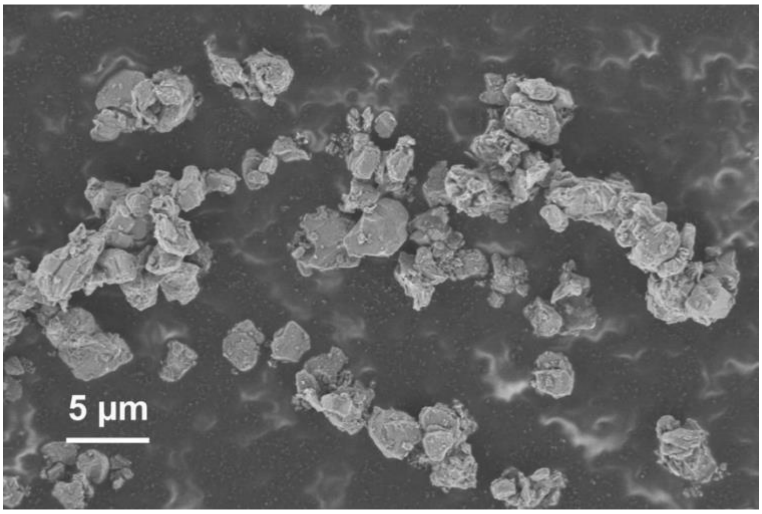

Figure S14. SEM image for P2-SC-BM, a mild mechanically ground product of P2-SC (300 mg of materials ball-milled for 20 min at 20 Hz using a Fritsch Pulverisette 23 Mini Mill with two zirconia balls (3 g each)), giving a more deagglomerated morphology. The SEM image was acquired by ThermoFischer Scientific Phenom ProX.
